# Supplementary material for: Food Next Door: From Food Literacy to Citizenship on a College Campus
Source: Int J Environ Res Public Health. 2021 Jan 11;18(2):534. doi: 10.3390/ijerph18020534 (PMC7827084; doi:10.3390/ijerph18020534)
Supplement: Supplementary file 1 [file ijerph-18-00534-s001.pdf]

The demographic portion of the survey was removed. For questions on the survey please contact nmeyer2@uccs.edu

Food Literacy

Please choose the best answer from the following options.

1. Do you know what a Community Supported Agriculture (CSA) is?
- Yes
- No
- I don't know

2. Which of the following food items is NOT commonly part of a CSA share?
- Vegetables
- Meat
- Pasta
- I don't know

3. In which season is butternut squash commonly harvested?
- Spring
- Summer
- Fall
- I don't know

4. In which season are tomatoes typically harvested?
- Spring
- Summer
- Fall
- I don't know

5. In which season is zucchini typically harvested?
- Spring
- Summer
- Fall
- I don't know

6. Where do asparagus naturally grow?
- Under the ground
- Above the ground
- On a tree
- I don't know

7. Where does a garlic (bulb) naturally grow?
- Under the ground
- Above the ground
- On a tree
- I don't know

8. Where do cherries naturally grow?
- Under the ground
- Above the ground
- On a tree
- I don't know

9. On average, what percentage of their income do Americans spend on food?

- o 10%
- o 20%
- o 30%
- o I don't know

10. On average, how many miles does food travel from harvest to consumer?

- o 3000miles
- o 1500miles
- o 500miles
- o I don't know

11. The more fish we eat, the less will be available for those living in coastal regions who depend on it for survival.

- o True
- o False
- o I don't know

12. Since the beginning of agriculture, biodiversity of edible plants has increased by 50%.

- o True
- o False
- o I don't know

13. Eating a diet rich in biodiversity improves nutrition and health.

- o True
- o False
- o I don't know

14. How much food is wasted in the U.S. annually?

- o 60%
- o 40%
- o 10%
- o I don't know

15. How many pounds of grains are needed to produce 1 lb. (pound) of conventionally raised beef?

- o 4lbs
- o 8lbs
- o 16lbs
- o I don't know

16. 30% of all carbon emissions come from agriculture.

- o True
- o False
- o I don't know

17. A supermarket is an example of a local food store.

- o True
- o False
- o I don't know

18. Of the following markets, which uses the least amount of energy?

- o Local grocery store
- o Chain grocery store
- o Farmers market
- o I don't know

19. Which of the following farming techniques is expected to deplete nutrients from the soil the fastest?

- Monoculture
- Polyculture
- Permaculture
- I don't know

20. Going to a farmers market has no effect on the local economy.

- True
- False
- I don't know

21. Do you know what a genetically modified organism (GMO) is?

- Yes
- No
- I don't know

22. In the U.S., which of the following is mostly a non GMO crop?

- Corn
- Carrots
- Soy
- I don't know

23. Do you know what a carbon footprint is?

- Yes
- No

24. Which of the following strategies has the smallest impact on our carbon footprint?

- Eating less meat
- Eating locally
- Eating organically
- I don't know

25. Which of the following protein sources has the lowest carbon footprint and the highest potential to boost health?

- Beef
- Cheese
- Beans
- I don't know

26. Have you heard of the Farm Bill?

- Yes
- No
- I don't know

27. Cucumbers can be best kept through fall and winter by which of the following?

- Freezing
- Pickling
- Drying
- I don't know

28. Which of the following does NOT celebrate food tradition?

- Attending a cultural food festival
- Eating a frozen pizza in front of the TV
- Cooking and eating together
- I don't know

29. Family meals can boost academic performance, nutrition and quality of life.
- o True
- o False
- o I don't know

30. Gardening or farming are low-intensity activities and not strenuous enough to improve fitness.
- o True
- o False
- o I don't know

31. Given the following options of locally and humanely raised animal proteins, which of them is the most sustainable choice in your diet?
- o Grass-fed beef
- o Heritage turkey
- o Local eggs
- o I don't know

32. Eating more vegetarian and vegan meals has no impact on one's health or sustainability.
- o True
- o False
- o I don't know

33. When kids grow vegetables, they eat more vegetables.
- o True
- o False
- o I don't know

Thank you very much for completing this survey.
